# Vitamin-mediated interaction between the gut microbiome and mitochondria in depression: A systematic review-based integrated perspective

**DOI:** 10.1016/j.bbih.2024.100790

**Published:** 2024-05-10

**Authors:** Ellen Bisle, Suchithra Varadarajan, Iris-Tatjana Kolassa

**Affiliations:** Department of Clinical & Biological Psychology, Institute of Psychology & Education, Ulm University, Albert-Einstein-Allee 47, 89081, Ulm, Germany

**Keywords:** Depression, Mitochondria, Microbiome, Vitamins

## Abstract

Depression is one of the world’s most prevalent mental disorders and its treatment remains suboptimal. Depression is a systemic disease with highly complex biological mechanisms. Emerging evidence points towards the involvement of mitochondria, microbiome and vitamins in its pathophysiology. Mitochondrial energy production was shown to be lowered in patients with depression. Mitochondrial energy production depends on vitamins, which are available from food, but are also synthesized by the gut microbiota. Several studies reported altered vitamin levels as well as changes in the gut microbiome composition and its vitamin metabolism in patients with depression. Therefore, the question of a connection between mitochondria and gut microbiome and vitamins influencing the mental health arises. This review aims to systematically investigate a combination of the topics – depression, mitochondria, microbiome, and vitamins – to generate an overview of a novel yet extremely complex and interconnected research field. A systematic literature search yielded 34 articles, and the results were summarized and bundled to develop this new integrative perspective on mitochondrial function mediated by the microbiome and microbiome-derived vitamins in depression. Furthermore, by discussing the research gaps this review aims to encourage innovative research approaches to better understand the biology of depression, which could result in optimized therapeutic approaches.

## Highlights

• Systematic review including mitochondria, microbiome and vitamins in the context of depression.
• Well-functioning mitochondrial energy production is vital for mental health.
• Microbiome daily produces many essential vitamins such as all B vitamins, vitamin C, K and metabolizes vitamin A.
• (Microbiome derived) vitamins could influence mitochondrial energy production.

## Graphical abstract

Evidence is pointing to an essential role of vitamins in the interaction of the gut microbiome and mitochondrial energy production in depression. Vitamin abundance is influenced by the food-derived nutrition uptake, but can be additionally produced by the gut bacteria. Food-derived prebiotics beneficially influence the gut microbiome, while food-derived antioxidants support the mitochondrial function, by preventing excessive ROS production. In future add-on treatments like dietary interventions, prebiotics, probiotics, or the use of supplements like vitamins to increase mitochondrial energy production, could help to positively increase vitamin availability, improve the gut microbiome and mitochondrial function and thereby advance therapy for depression.

**Figure:**
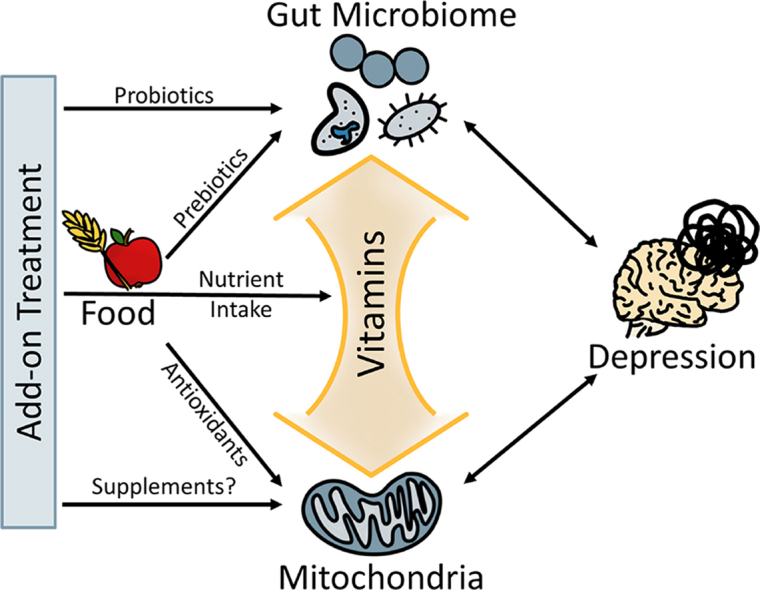

## 1 Introduction

Major depressive disorder (MDD) is a leading cause of disability, affecting approximately 280 million people worldwide (Olesen et al., 2012; World Health Organization, 2021). Along with the human suffering, treatment of depression incurs a high socioeconomic cost, for e.g. it costs 90 billion € per year in Europe itself (Olesen et al., 2012). Irrespective of the several existing theories on disease development, the precise biological mechanisms of MDD remain enigmatic. Moreover, current treatment approaches like psychotherapy and psychopharmacology report high treatment resistance and relapse rates (Al-Harbi, 2012; Insel and Wang, 2009). Thus, there is a pressing need to gain a more holistic view on the heterogeneity of the disease. In this line, transdisciplinary research provided evidence for various far-reaching biological changes in the metabolism of MDD patients, such as reduced mitochondrial energy production (Gardner, 2003; Karabatsiakis et al., 2014; Zvěřová et al., 2019), increased inflammation, oxidative stress (Haapakoski et al., 2015; Palta et al., 2014), and alterations in the gut microbiome (McGuinness et al., 2022; Simpson et al., 2021). Taken together, this evidence indicates systemic biological, in particular immunometabolic, alterations in MDD.

### 1.1 Mitochondrial energy production in depression

Until recently mental illnesses have been viewed as brain-related diseases, caused by changes in neurotransmitter signaling (Filatova et al., 2021). However, emerging evidence suggests a paradigm shift from this view. The brain demands approximately one fifth of the body’s energy needs (Shulman et al., 2004). Even at resting state the brain consumes energy for processes like maintaining neurons’ membrane potentials, packing neurotransmitters in vesicles, releasing neurotransmitters and moving receptors and ion channels to and from the cell surface, etc. (Gleichmann and Mattson, 2011; MacAskill et al., 2010; Shulman et al., 2004). Mitochondria are responsible for the majority of energy production in the cells (Wilson, 2017); they break down energy-rich nutrients like sugars, amino acids or fatty acids, and thereby – in a process called oxidative phosphorylation (OXPHOS) – produce the cells’ energy, namely adenosine triphosphate (ATP). OXPHOS is located at the inner mitochondrial membrane, and oxygen is needed as an electron and proton acceptor during ATP production. Without mitochondrial ATP, neurons are not able to keep their function and die rapidly as they lack the important glycolytic enzyme 6-phosphofructo-2-kinase and cannot produce ATP from glycolysis (Bolaños et al., 2010). This underlines the importance of well-functioning mitochondria in the brain.

Previous studies show that the energy metabolism and especially OXPHOS is altered in depression, which is seen in blood (PBMC and platelets; Karabatsiakis et al., 2014; Zvěřová et al., 2019) and muscle cells (Gardner, 2003). In addition, reduced ATP levels and reduced glucose consumption can be observed in individuals with depression (Baxter, 1989; Moretti et al., 2013). During OXPHOS an inevitable production of incompletely reduced oxygen takes place (Mazat et al., 2020), which is the source of reactive oxygen species (ROS). To prevent excessive ROS levels, vitamins and enzymes like the superoxide dismutase, or the glutathione system enable the cells to lower their own ROS levels (Grimm and Eckert, 2017). Imbalances between ROS production and depletion, e.g. when antioxidative defense is impaired and/or the ROS production is too high, make cells suffer from oxidative stress (Liu et al., 2015). Oxidative stress is associated with depression (Black et al., 2015; Jiménez-Fernández et al., 2022). ROS production is higher during inflammation and it is shown that anti-inflammatory treatment can improve recovery of patients with depression, while higher inflammation levels are known as a risk factor for developing depression (Haapakoski et al., 2015; Palta et al., 2014; Peirce and Alviña, 2019). Concomitantly, this evidence points towards the crucial role of mitochondrial energy production in major depression.

### 1.2 The pivotal role of vitamins in mitochondrial energy production, depression, and their interconnection

The citrate cycle is the central metabolic pathway degrading metabolic intermediates of glucose, fatty acids and proteins. In this process nutrients are finally broken down to carbon dioxide, electrons and protons which are transferred to Flavin Adenine Dinucleotide (FAD^+^) and Nicotinamide Adenine Dinucleotide (NAD^+^). During mitochondrial OXPHOS, these electrons are cascading across the mitochondrial complexes, till they are transferred to oxygen at complex IV, forming water. By transferring electrons, the complexes pump protons into the intermembrane space of the mitochondria. This generates a proton gradient that is used to generate ATP (Nolfi-Donegan et al., 2020). The energy production is dependent on vitamins, as B-complex vitamins are important cofactors of the OXPHOS and therefore essential for an unimpaired function of the OXPHOS and citrate cycle (as reviewed by Wesselink et al., 2019). Vitamin B1, B5 and B12 are needed in the citrate cycle, complex I and II activity are supported by vitamin B2 and E, complex III and IV by vitamin E. Finally, vitamin B2 and B3 are precursors of FAD^+^ and NAD^+^, needed for the proton and electron transfer into the respiratory chain (Wesselink et al., 2019). Apart from their direct support of the OXPHOS, vitamins like vitamin C and E can reduce oxidative stress due to their antioxidant capacity (Carr and Maggini, 2017; Lee et al., 2022).

Several studies reported lowered vitamin intake or vitamin concentrations in the body in association with MDD (Al-Fartusie et al., 2019; Hoang et al., 2011; Miškulin et al., 2014; Nguyen et al., 2017; Prohan et al., 2014; Tolmunen et al., 2003). Even antidepressant pharmacotherapy success could be predicted by serum vitamin B9 levels, showing lowered success with lowered vitamin levels (Fava et al., 1997). These results provide strong evidence on the importance of vitamins in depression.

### 1.3 The gut microbiome in depression

There is evidence pointing to a connection between the gut microbiome composition and our brain, via the so called (microbiome-) gut-brain axis. Any perturbations in the gut-brain axis can lead to various mental and neurodegenerative diseases (Dinan and Cryan, 2017).

Studies have already reported alterations in the gut microbiome composition of patients with depression. For instance, even though results have been quite variable between studies, depression has been associated with a decreased abundance of *Bacteroidetes* and *Prevotellaceae* and an increased abundance of *Actinobacteria*, *Enterobacteriaceae*, *Bifidobacteriaceae* and *Lachnospiraceae* (Simpson et al., 2021). Further, the gut microbiome could play a decisive role in the etiology of MDD, by providing our body with multiple metabolites which have antioxidant, mitochondria-/and immune-regulatory properties (Agirman et al., 2021; Aguilar-López et al., 2020; Rudzki et al., 2021). This available evidence leads to the postulation that the microbiome and its metabolome could be important modulators of MDD.

### 1.4 Vitamins – a potential connector between gut microbiome and mitochondrial energy production in depression

Besides vitamin shortage, mitochondrial function can be altered by gut microbiome-derived metabolites. However, at present the microbiome’s vitamin production capacity is receiving less research attention. Hence, it is essential to derive advanced knowledge on the role of vitamins, as it could be an important connection between microbiome and mitochondrial function in the context of mental health.

The human body cannot produce vitamins and is therefore reliant on vitamins derived from food or produced by the gut microbiome (Hill, 1997). The gut microbiome has shown the potential to produce a variety of vitamins, namely vitamin B1, B2, B3, B5, B6, B7, B9, B12, C and K (Chang et al., 2019; Hill, 1997; Rudzki et al., 2021). The microbial vitamin production might not be sufficient to cover the body’s daily needs, but for vitamin B6, B9 and B12 gut bacteria can produce more than 30% of the daily reference intake (Rudzki et al., 2021). The spectrum of bacteria-producing vitamins is broad, and different enterotypes are associated with distinct potential for vitamin synthesis (Arumugam et al., 2011). Notably, depressed individuals show higher abundance of the *Bacteroides* enterotype subtype 2, which was characterized by reduced *Faecalibacterium* and lower microbial load and might therefore be a dysfunctional subgroup of the *Bacteroides* enterotype (Valles-Colomer et al., 2019). Taken together there is a strong indication pointing towards a connection of microbiome, vitamins, and mitochondria in the course of MDD.

This review focuses on providing a systematic overview on the studies regarding microbiome, mitochondria, and vitamins in depression. Further aims to address open research questions on mitochondria and microbiome connection, which could be mediated by vitamins in depression.

## 2 Methods

### 2.1 Search strategy

A systematic literature search was performed in PubMed, Embase (Ovid), Medline (Ovid), Web of Science and Google Scholar by applying PRISMA guidelines (Page et al., 2021) between May 2022 and April 2023. The search included articles in both German or English language.

Based on the recommendation by Muka et al. (2020) that the first 200 results from Google Scholar search delivers the best results, we included the first 200 results. The search captured human or rodent studies which involved the four main topics of this review (depression, microbiome, mitochondria and vitamins). Following a PICO scheme, the patient population were depressed individuals or rodents expressing depressive-like behavior, either receiving no intervention or being treated with vitamin or probiotic supplementation. Comparisons were supposed to be drawn between the depressed group and healthy controls or treatment and placebo group. Outcome measures considered were mitochondrial function or morphology, oxidative phosphorylation, cellular energy production, oxidative stress, serum/blood/cellular vitamin levels or vitamin intake and (stool) microbiome abundance.

Our initial literature research could not yield any original research results which included all four topics within a single study. To address this challenge, the further literature search was split into four research sections, each section consisting of three of the four main topics, thus making it possible to elucidate the research gap from different perspectives. The research sections were:

i) depression, microbiome, and mitochondria,
ii) depression, mitochondria, and vitamin,
iii) depression, microbiome, and vitamin, and
iv) vitamin, microbiome, and mitochondria

An example of the research strategy in PubMed is provided in Table 1.

**Table 1:** Search strategy in PubMed.

Database	Parts	Topics	Search strategy
PubMed	1	D, Ba, Mi	((((("Depressive Disorder"[mh])) AND (((("Mitochondria"[Mesh] OR "Mitochondrial Size"[Mesh]) OR "Oxidative Phosphorylation"[Mesh]) OR "Energy Metabolism"[Mesh]))) AND ((("Brain-Gut Axis"[Mesh] OR "Microbiota"[Mesh] OR "Gastrointestinal Microbiome"[Mesh]) OR "Fecal Microbiota Transplantation"[Mesh])))))
	2	D, Mi, V	((((((("Depressive Disorder"[mh])) AND (("Vitamins"[Mesh] OR "Vitamin B Complex"[Mesh] OR "Vitamin K"[Mesh] OR "Vitamin E"[Mesh] OR "Vitamin D"[Mesh] OR "Vitamin A"[Mesh] OR "Vitamin B 12 Deficiency"[Mesh]))) AND (((("Mitochondria"[Mesh] OR "Mitochondrial Size"[Mesh]) OR "Oxidative Phosphorylation"[Mesh]) OR "Energy Metabolism"[Mesh]))))
	3	D, Ba, V	((((("Depressive Disorder"[mh])) AND (("Vitamins"[Mesh] OR "Vitamin B Complex"[Mesh] OR "Vitamin K"[Mesh] OR "Vitamin E"[Mesh] OR "Vitamin D"[Mesh] OR "Vitamin A"[Mesh] OR "Vitamin B 12 Deficiency"[Mesh]))) AND ((("Brain-Gut Axis"[Mesh] OR "Microbiota"[Mesh] OR "Gastrointestinal Microbiome"[Mesh]) OR "Fecal Microbiota Transplantation"[Mesh])))))
	4	V, Ba, Mi	((((("Vitamins"[Mesh] OR "Vitamin B Complex"[Mesh] OR "Vitamin K"[Mesh] OR "Vitamin E"[Mesh] OR "Vitamin D"[Mesh] OR "Vitamin A"[Mesh] OR "Vitamin B 12 Deficiency"[Mesh])) AND (((("Mitochondria"[Mesh] OR "Mitochondrial Size"[Mesh]) OR "Oxidative Phosphorylation"[Mesh]) OR "Energy Metabolism"[Mesh]))) AND ((("Brain-Gut Axis"[Mesh] OR "Microbiota"[Mesh] OR "Gastrointestinal Microbiome"[Mesh]) OR "Fecal Microbiota Transplantation"[Mesh])))))
		final	1 OR 2 OR 3 OR 4

*Note*: Depression (D), Microbiome (Ba), Mitochondria (Mi), Vitamins (V).

### 2.2 Inclusion and exclusion criteria

Studies which included research on depression (research part i, ii and iii) had to assess depression using questionnaires/interviews in human studies or behavioral tests in rodent models. Human study participants had to be 18 years or above for study inclusion. For studies including mitochondria (research part i, ii and iv) the following measurements were included: respiratory function, morphology, gene expression or oxidative stress. For studies including the microbiome (research part i, iii and iv), bacterial composition had to be measured by using DNA sequencing techniques or probiotic treatment had to be applied. For inclusion of studies with vitamins (research parts ii, iii, and iv), studies needed to assess vitamin intake or concentration of vitamins in the body or perform vitamin supplementation. Studies were excluded if they were performed in plants, insects, birds, mammals (except rodents) or investigated less than three of the main topics. Literature search results were independently screened for title and abstract and in a second step for whole text. In the animal studies risk of bias was assessed by applying systematic review centre for laboratory animal experimentation (SYRCLE) criteria (Hooijmans et al., 2014) (see Supplementary Table 1).

### 2.3 Data extraction

Included studies were individually screened and data on study population, intervention time, number of participants/animals, assessment strategies (for depression, microbiome, mitochondria and vitamins), information about intervention, results for the three main topics of the research part was tabularly assessed.

## 3 Results

**Fig. 1:**
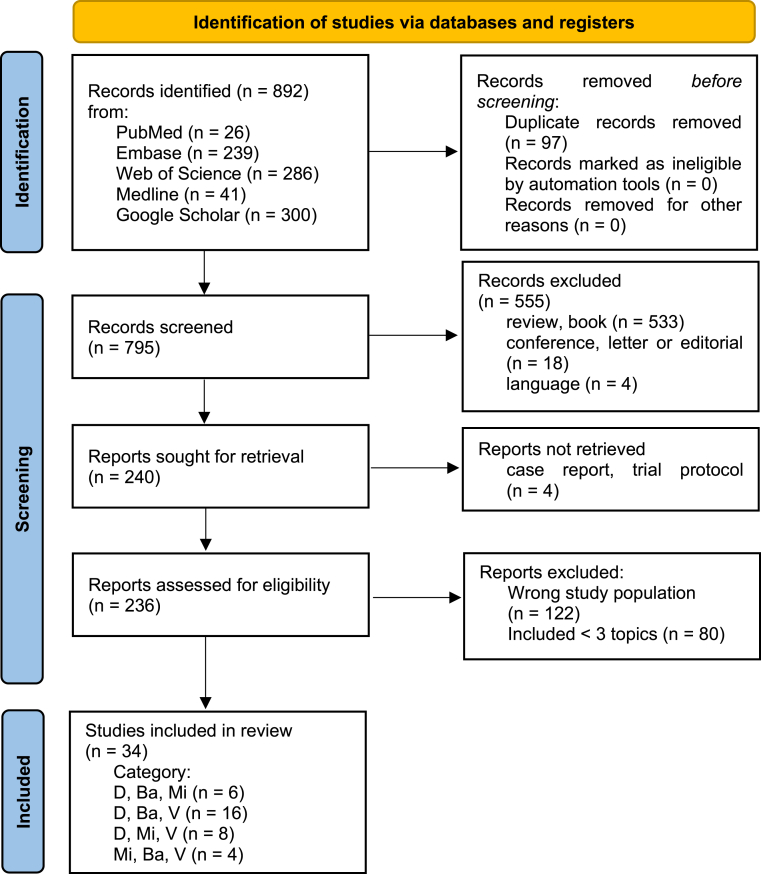
Flowchart of literature research and selection *Annotation*s: depression (D), microbiome (Ba), mitochondria (Mi), vitamins (V). This figure was designed based on the PRISMA guidelines (Page et al., 2021). No automated tools were used for record exclusion.

A total of 892 results were found by literature search. After duplicate depletion 795 articles were left. After application of exclusion criteria 34 articles were selected for this review (see Fig. 1). All articles covered three of the four topics of interest (depression, microbiome, mitochondria and vitamins), but no article included all four topics. A total of 16 animal studies and 18 studies with human participants were included.

### 3.1 Interconnection between gut microbiome and mitochondria in depression

The literature search revealed a total of 4 animal and 2 human studies investigating the connection between microbiome, mitochondria and MDD (see Table 2 and Table 3). Animal studies showed that animals receiving fecal microbiome transplantation (FMT) from MDD patients showed not only more depressive-like behavior (Liu et al., 2021; S. Liu et al., 2020), but also showed different hippocampal protein acetylation and succinylation with approximately 5% of proteins involved in mitochondrial processes like glycolysis/gluconeogenesis, the citrate cycle, biosynthesis of amino acids, and OXPHOS compared to animals receiving FMT from healthy controls (Liu et al., 2021). Furthermore, mitochondria of intestinal cells of rats receiving FMT from MDD patients looked swollen and showed vacuoles, which were absent in rats receiving FMT from healthy controls or without FMT (S. Liu et al., 2020).

**Table 2:** Depression, Microbiome and Mitochondria in rodents.

Authors	Study population//Intervention time//n		Depression	Microbiome	Mitochondria
Liu et al. (2021)	Male GF Kunming mice (age: 8 weeks)//One day of FMT//Behavioral experiment: n = 11 MDD receiver mice; n = 10 HC receiver mice. Brain extraction: n = 6 of each group.	M/I	FST two weeks after FMT	FMT form human (MDD, healthy) in germ free mice.	Assessment of acetylated or succinylated lysin in hippocampus.
		R	More immobility of MDD receiver mice.	–	High percentage of acetylated or succinylated proteins and affected pathways in mitochondria. Succinylated proteins showed 3 clusters: ribosomes, endocytosis and oxidative phosphorylation.
S. Liu et al. (2020)	GF rats (age: 8 weeks)//2 weeks//n = 18 in 3 groups (MDD microbiome, HC microbiome, blank control)	M/I	Human donors: HAMD >24 points; no antidepressants./Rats: FST (after 4 weeks). SPT (after 1, 2, 3 and 4 weeks).	FMT form human (MDD, healthy) to rat (once daily for 2 weeks).	Morphology, transmission electron microscope.
		R	In MDD receiver rats FST: more immobility and SPT was lowered.	–	Mitochondria in intestinal epithelial cells of MDD receiver rats showed abnormal appearance. In blank and healthy control group normal mitochondrial appearance.
Lukić et al. (2019)	Male mice BALB/c (age: 8–10 weeks)//3 weeks, daily i.p. injection//n = 160 in 15 groups	M/I	TST, FST, SPT; Antidepressant application (in mg/kg) with/without bacterial combination: control (PBS), fluoxetine (10), escitalopram (10), venlafaxine (10), duloxetine (dul, 10), desipramine (20); *Ruminococcus* (Rum, 10^9^ CFU): control, dul, Rum, dul + Rum, *Adlercreuzia* (Adl, 10^9^ CFU): control, dul, Adl, dul + Adl.	16S rRNA (V4)	Gene expression in the mPFC. Only done in *Ruminococcus* groups but not for *Adlercreutzia* groups.
		R	Antidepressants led to improvements in FST and SPT. With *Ruminococcus* the antidepressant effect was lower, but *Adlercreutzia* had no effect on depressive tests.	Antidepressant treatment resulted in lowered *Ruminococcus* and *Adlercreutzia* abundance in stool samples.	*Ruminococcus flavefaciens* results in increased mitochondrial gene expression and oxidative phosphorylation related genes (complex I and III).
Wang et al. (2022)	Male mice C57BL/6 (age: 8–10 weeks)//35 days CUMS//n = 40 in 4 groups (control, CUMS, CUMS + tryptophan, tryptophan) for RNA-sequencing n = 6 per group	M/I	CUMS; SPT, TST	16S rRNA (V3–V4), gut tissue staining, gene expression.	Gene expression (brain).
		R	CUMS group compared to control: significantly lower bodyweight, lower SPT, longer immobility in TST. Tryptophan supplementation reversed effects of CUMS.	CUMS as well as tryptophan treatment altered the microbiome. CUMS group compared to control: significant increase of *Escherichia* and *Akkermansia muciniphila*, increased serum LPS is, reduced mRNA and protein abundance of Claudin-1 and ZO-1. Tryptophan supplementation reversed effects of CUMS.	CUMS group compared to control: reduced OXPHOS involved genes, altered nicotinamide metabolism. Tryptophan supplementation reversed effects of CUMS for some genes.

Note: Articles included after application of inclusion and exclusion criteria on systematic literature search results. Abbreviation: Chronic Unpredictable Mild Stress (CUMS); Faecal Microbiota Transplantation (FMT), Germ Free (GF), Colony Forming Units (CFU), Forced Swim Test (FST), Oxidative Phosphorylation (OXPHOS), Sucrose Preference Test (SPT), Tail Suspension Test (TST), Major Depressive Disorder (MDD), Measurement/Intervention (M/I), Result (R).

**Table 3:** Depression, Microbiome and Mitochondria in humans.

Authors	Study population//Intervention time//n		Depression	Microbiome	Mitochondria
Akkasheh et al. (2016)	MDD patients (score of >15 on the 17-item HAMD); age 20–55 years (mean 37.25(Placebo: 36.2; Probiotic: 38.3))//8 weeks once daily//n = 40, 20 per group (17 female, 3 male)	M/I	MDD diagnosis according to DSM-IV criteria. Severity of depressive symptoms assessed by BDI.	Probiotic or placebo (starch): *Lactobacillus acidophilus* (2x10ˆ9 CFU/g), *Lactobacillus casei* (2x10ˆ9 CFU/g), and *Bifidobacterium bifidum* (2x10ˆ9 CFU/g).	GSH levels, total antioxidant capacity (measured with the Ferric Reducing Ability of Plasma [FRAP]).
		R	BDI lower in probiotic group compared to placebo.	–	Higher GSH in probiotic vs placebo group but effect was lost after controlling for fasting plasma glucose, age and BMI. Decreased CRP in probiotic group. No difference in total antioxidant capacity.
Venturini et al. (2019)	Patients with CFS/ME diagnosis (age 30–40 years)//8 weeks probiotic//n = 9	M/I	Severity of depressive symptoms assessed by BDI.	Alternating use of: *Enterococcus faecium, Saccharomyces boulardii; B. longum, B. breve, B. bifidum,* and *B. infantis; B. longum; L. casei* and *B. lactis; L. rhamnosus and L. acidophilus.*	ROS production: photometrically tested in heparinized plasma.
		R	Reduced BDI after 8 weeks of probiotic treatment.	–	After 8 Weeks of probiotic treatment: ROS reduced in people with normal baseline levels, but ROS increased if people had low baseline levels.

Note: Articles included after application of inclusion and exclusion criteria on systematic literature search results. Abbreviation: Beck Depression Inventory (BDI), Diagnostic and Statistical Manual of Mental Disorders 4^th^ version (DSM-IV), Hamilton Depression Scale (HAMD), Glutathione (GSH), Colony Forming Units (CFU), Measurement/Intervention (M/I), Result (R), Reactive Oxygen Species (ROS).

Lukić et al. (2019) treated healthy mice with different antidepressant combinations, resulting in lowered immobility in the forced swim test (FST), increased sucrose preference and reduced abundance of *Ruminococcus* and *Adlercreutzia* in fecal samples. Application of duloxetine in combination with *Ruminococcus flavefaciens* abolished antidepressant effects, while increasing mitochondrial gene expression in the medial prefrontal cortex and positively correlating with mitochondrial complex I, III and mitochondrial ribosomal protein (Lukić et al., 2019). Wang et al. (2022) applied chronic unpredictable mild stress on mice, resulting in a downregulation of OXPHOS genes in the brain, altered gut microbiome composition (e.g. increased *Escherichia* and *Akkermansia muciniphila*), increased serum LPS values and higher depressive-like behavior (Wang et al., 2022). Thus, the rodent study results demonstrate a connection between the microbiome and (brain) mitochondrial morphology and biochemistry.

Looking at the human studies included, patients with depressive symptoms were provided with an 8-week probiotic treatment (see Table 3), which significantly decreased depressive symptoms (Akkasheh et al., 2016; Venturini et al., 2019). The total antioxidant capacity did not differ between probiotic and placebo group in MDD patients (Akkasheh et al., 2016). Here mitochondria were not directly assessed, but as mitochondria are the main producers of oxidative stress in form of ROS (Mazat et al., 2020), an assumption on mitochondrial involvement could be drawn. In patients with chronic fatigue syndrome/myalgic encephalomyelitis, lowered blood ROS metabolites were only reported for patients showing normal ROS levels before the 8-week probiotic treatment. In contrast, patients having lower levels before treatment showed an increase in ROS metabolites (Venturini et al., 2019).

To summarize, the literature search results supported a microbiome-mitochondria connection linked to mental health. Nevertheless, it is still unclear with which proportion and via which communication pathways this connection is mediated. One such connectivity option between the gut and mental health could be through (microbiome-derived) vitamins.

### 3.2 Interconnection between gut microbiome and vitamins in depression

To address the hypothesis of a vitamin-mediated improvement of depressive symptoms by the gut microbiome, this review included articles with 4 animal and 12 human studies (see Table 4 and Table 5).

**Table 4:** Depression, Microbiome and Vitamins in rodents.

Authors	Study population//Intervention time//n		Depression	Microbiome	Vitamin
Hao et al. (2021)	Male C57BL/6 mice (age: 8 weeks)//12 weeks: fist 8 weeks chronic unpredictable mild stress, last 4 weeks with or without treatment with coniferyl ferulate (CF)//n = 45 (three groups: control, CUMS with/without CF treatment)	M/I	SPT, FST, TST	16S rRNA (V3–V4)	Mass spectrometry
		R	CF lowered depression after CUMS compared to CUMS group without CF treatment.	CF reduces CUMS induced morphology changes and inflammation in colon. CF rebuilds the microbiome of CUMS mice. CUMS significantly reduced *Lactobacillus*, which was improved by CF. *Allobaculum* was significantly increased in the CF group. The CF group had significantly less *Klebsiella, Bacteroides* and *Enerobacteriaceae*.	Thiamine, ascorbate, B6, metabolism in gut microbiome is enriched after the CF treatment. *Lactobacillus* abundance correlates positively with enriched metabolites in the CF group.
Jiang et al. (2020)	Male C57BL/6J mice (age: 7 weeks)//10 weeks alcohol exposure, daily administration of Nicotinamide Riboside (NR). FMT from experimental mice to receiver mice for 2 weeks.//Experimental mice: n = 21 (3 groups: Control, alcohol exposure, alcohol exposure + NR); FMT receiver mice: n = 18 (same groups as experimental)	M/I	SPT, FST, EPM, Y-maze	16S rRNA (V3–V4), FMT	NR 400 mg/Kg
		R	NR reduced alcohol-induced depressive symptoms and made it more comparable to control group.	Lowered in *Prevotella, Barnesiella, Alloprevotella* and *Alistipes*, while *Akkermansia* was significantly enriched. NR treated group had no difference compared to control. At the genus level *Akkermansia* and *Clostridium XVIII* were the two most abundant bacteria in the alcohol group, while *Barnesiella* and *Alloprevotella* were the most abundant in the NR group. After FMT the receiver mice showed similar depressed behavior like donor mice.	–
Song et al. (2019)	Male Wistar rats (age: 10 weeks)//14 days ACTH injection (100 μg)//n = 20 (control and ACTH group)	M/I	Induced depression by ACTH injection.	16S rRNA (V3–V4)	–
		R	ACTH injection leads to depression-like symptoms.	*Ruminococcus* and *Klebsiella*, *Anaeroplasma*, *Anaeroplasmataceae*, *Anaeroplasmatales* and *Oscillospira* higher in ACTH group; *Lactobacillus* and *Akkermansia*, *Lactobacillaceae*, *Coprococcus*, *Actinobacteria*, *Actinomycetales*, *Lachnobacterium* and *Burkholderiales* were lower.	ACTH treatment changed Vitamin C pathways and significantly lowered Vitamin C abundance was seen in urine.
M. Zhang et al. (2023)	Male ICR mice (18–22g)//Six weeks CUMS, within the last 14 days antidepressant treatment//n = 24 (four groups: control, CUMS, CUMS with imipramine, CUMS with Matrine)	M/I	CUMS, SPT, FST	16S rRNA (V3–V4)	Metabolome analysis
		R	CUMS induced depressive-like symptoms in all tests, which could be alleviated by both treatments applied.	Compared to control, *Lactobacillus* and *Allobaculum* were decreased, while *Rikenella* and *Odoribacter* were increased in the CUMS cohort. Antidepressant treatment lowered *Rikenella, Odoribacter, Ruminococcus* and *Helicobacter* and increased *Lactobacillus*.	Vitamin digestion and absorption was altered in CUMS, which could be (partially) restored by antidepressant treatment.

Note: Articles included after application of inclusion and exclusion criteria on systematic literature search results. Abbreviation: Adrenocorticotropic Hormone (ACTH), Coniferyl Ferulate (CF), Colony Forming Units (CFU), Chronic Unpredictable Mild Stress (CUMS), Faecal Microbiota Transplantation (FMT), Forced Swim Test (FST), Measurement/Intervention (M/I), Nicotinamide Riboside (NR), Result (R), Sucrose Preference Test (SPT), Tail Suspension Test (TST).

**Table 5:** Depression, Microbiome and Vitamins in humans.

Authors	Study population//Intervention time//n		Depression	Microbiome	Vitamin
Ciocan et al. (2021)	MDD patients (mean age (years): MDD 42, healthy controls 42)//3 months antidepressant treatment//n = 112 healthy controls, n = 56 MDD patients (venlafaxine (n = 25), citalopram (n = 19), escitalopram (n = 12))	M/I	MDD diagnosis according to DSM-IV. Treatment response: >50% reduction of HDRS after 3 months.	16S rDNA	Metabolome analysis
		R	57% responded to the 3 months treatment.	MDD patients had a lower relative DNA abundance of *Fusobacteria, Saccharibacteria, Actinomyces, Flavobacterium, Enterococcus, Neisseria, Tepidimonas, Aggregatibacter, Curvibacter, Fusobacterium* in blood. While *Kocuria, Chryseobacterium, Parvimonas* and *Janthinobacterium* were enriched in MDD patients. Treatment response was associated with high *Firmicutes* and low *Bosea, Tetrasphaera* abundance before medication start.	Treatment responders had lower retinol levels, non-responders lower riboflavin, nicotinate and nicotinamide levels in blood.
Duan et al. (2022)	Healthy men (age matched groups, 20–60 years)//N = 31 medically healthy imprisoned persons, N = 30 healthy not imprisoned	M/I	Severity of depressive symptoms assessed by SDS.	16S rRNA	–
		R	SDS did not reach the threshold of clinically significant depression.	Lowered alpha diversity in prisoners. Relative abundance of *Prevotella* decreased and of *Bacteroides* increased in prisoners. SDS negatively correlated with *Bacteroidetes, Anaerotruncus, Prevotella*. Positive correlation of SDS with *Coprobacillus*. SDS symptoms severity (high vs low) showed a clear PCA separation within groups.	Lower expression of microbiome genes involved in vitamin metabolism in imprisoned persons.
Feher et al. (2014)	Patients with ocular dysesthesia and hyperesthesia showing comorbid anxiety and depression symptoms (mean age (years): vitamin and probiotic treatment 45, vitamin treatment 46)//8-week intervention, 3 times daily//n = 60 (20 each group: healthy control, vitamin and probiotic treatment, vitamin treatment)	M/I	Severity of depressive symptoms assessed by Hospital anxiety and depression scale-based Questionnaire.	Probiotic treatment group: Brand name "Framelim" containing *Lactobacillus acidophilus* (1.25 × 109 CFU) and *Bifidobacterium longum* (1.35109 CFU), vitamin A (0.15 mg), vitamin D (1.08 mcg) and EPA (44 mg), DEA (58 mg) fatty acids, vitamins B1 (thiamin 0.5 mg), B3 (niacin 5 mg), B6 (pyridoxin 0.5 mg), B9 (folate 0.075 mg) and B12 (cyanocobalamin 0.5 mg).	See microbiome; vitamin treatment group: received same treatment, excluding bacterial lysates, vitamin A and D.
		R	Probiotic-vitamin mix lowered depression, while vitamin only did not reduce the depression symptoms.	–	–
Li et al. (2022)	Bipolar disorder (BD) patients with a depressive episode (mean age (years): BD 24, healthy controls 21)//n = 109 BD, n = 40 healthy controls	M/I	Severity of depressive symptoms assessed by HAMD and MADRS. HAMD score ≥14 were defined as presence of current depressive symptoms.	Whole genome sequencing	Metabolome analysis
		R	–	Relative abundance of vitamin B9 and B2 synthesizing microbiota is higher in healthy controls than in BD patients. BD patients showed enriched abundance of species of the *Streptococcaceae, Fusobacteriaceae, Tissierellaceae, Bacteroidaceae* and *Actinomycetaceae*. Lower abundance was seen in BD patients for *Akkermansiaceae, Yersiniaceae, Enterobacteriaceae, Acidaminococcaceae, Eubacteriaceae, Ruminococcaceae, Morganellaceae, Flavobacteriaceae.*	Vitamin B9, B5 and B2 and catabolic products of vitamin B6 correlated negatively with symptom severity. Vitamin B6 correlated positively with symptom severity.
Maitiniyazi et al. (2022)	Breast cancer patients (BCP) with or without depression (mean age (years): BCP without depression 54, BCP with depression 50)//Assessment of dietary habits with a 3 days dietary recall//n = 205 (n = 60 BCP with depression)	M/I	Severity of depressive symptoms assessed by CES-D.	16S rRNA (V3–V4) (n = 37 stool samples from BCP without depression, n = 18 from depressed patients).	HPLC
		R	CES-D scores are negatively correlated with Vitamin A, B2, niacin intake and α-diversity. Effects of the Chinese healthy eating index on the Depression score are mediated via the microbiome.	Significant alpha diversity reduction of depressed patients. Abundance of: *Clostridia, Firmicutes, Lachnospirales, Lachnospiraceae* and *Blautia* was higher in nondepressed patients. *Gammaproteobacteria, Proteobacteria, Enterobacteriaceae, Enterobacterales, Escherichia-Shigella* and an unidentified *Prevotellaceae* were higher in patients with depression.	*Campilobacterota* and *Streptococcus* were negatively correlated with B2 intake. *Streptococcus* negatively correlated with vitamin B3. Positive correlations were seen between vitamin B2 intake and *Caldatribacteriota* and *Ruminococcus* as well as for niacin intake and *Ruminococcus*.
Mayneris-Perxachs et al. (2022)	Persons with no, mild or severe depression (mean age (years): IRONMENT cohort; not depressed 53, mild depressed 51, severe depressed 47/IMAGEOMICS cohort; not depressed 68, mild depressed 67, major depressed 66)//human: no depression n = 44, mild depression n = 47, severe n = 25	M/I	Severity of depressive symptoms assessed by PHQ-9.	Whole genome sequencing	Food -frequency questionnaires
		R	–	Human: no alpha diversity difference (Shannon index). Participants with higher PHQ-9 scores at baseline had higher *Acidaminococcus, Murdochiella, Streptococcus, Parabacteroides, Clostridum carboxidivorans, C. lavalense* and *Lactobacillus pentous* abundance. Lowered abundance of *Syntrophomonas, Clostriduim ultuneense, Desulfotomaculum ruminis, Odoribacter, Butyrivibrio crossotus, Paenibacillus polymyxa, Bifidobacterium psoedolongum, Eubacterium, Alkaliphilus peptidifermentans, Parasporobacterium, Roseburia* (*hominis, intestinalis*). After the one-year follow-up higher PHQ-9 scores were associated with higher *Prevotella, Rikenella microfusus, Klebsiella, Vibro, Enterobacter, Shigella sonnei, Citrobacter* abundance. Lower abundance was seen for *Lachnospiraceae, Paramaledivibacter caminithermalis, syntrophomonas zhenderi, Sedimentibacter, Closrtidia, Bifidobacterium* (*cuniculi, boum, pseudocatenulatum, pseudolongum, gallicum, tsurumiense, pullorum*), *Leprotrichia hofstadii*.	Lowered microbiome derived Vitamin B1 and B7 metabolism pathways showed tendencies to lowered PHQ-9 scores. Positive correlation between Vitamin B1 intake as well as folate metabolism and PHQ9. Negative correlation between Vitamin E intake and PHQ9.
Raygan et al. (2018)	People with diabetes type 2 and coronary heart disease, (mean age (years): placebo 67, vitamin and probiotic treatment 72)//12 weeks treatment//n = 60 (n = 30 per group)	M/I	Severity of depressive symptoms assessed by BDI.	The treatment group received a total 8x19ˆ9 CFU/g containing equal parts of *Lactobacillus acidophilus, Bifidobacterium bifidum, Lactobacillus reuteri*, and *Lactobacillus fermentum* and vitamin D.	50,000 IU vitamin D every two weeks or placebo.
		R	Lower BDI scores after intervention in treatment group compared to placebo.	–	Higher vitamin D levels after treatment compared to placebo.
Reininghaus et al. (2020)	MDD patients (mean age (years): vitamin B7 and Probiotic treatment group 43, vitamin B7 treatment group 40)//28 days once daily vitamin B7 supplementation//n = 82 (2 groups: vitamin B7 treatment, vitamin B7 and probiotic treatment) Stool samples of 53 patients n = 26 probiotics, n = 27 placebo collected	M/I	Severity of depressive symptoms assessed by HAMD, the BDI-II and the Symptom Checklist-90-Revised (SCL-90). Study inclusion only with current ICD 10 F3 diagnosis.	16S rRNA (V3–V4), Probiotic treatment with “OMNi-BiOTiC® Stress Repair” including *B. bifidum W23, B. lactis W51, B. lactis W52, L. acidophilus W22, L. casei W56, L. paracasei W20, L. plantarum W62, L. salivarius W24 and L. lactis W19.*	125 mg D-Biotin (vitamin B7) given to both groups.
		R	No difference between treatment groups. Both groups reduced depressive symptoms.	No change in α-diversity between treatment groups and timepoints. Probiotic treatment increases *Ruminococcus gauvreauii* and *Coprococcus*.	Pathways of Vitamin B7, B6, B3, B1 metabolism and for Vitamin B5 and CoA biosynthesis were shown to be enriched in the probiotic treatment group.
Rhee et al. (2020)	Patients with MDD or BD or healthy controls (mean age (years): MDD 46, BD 34, healthy controls 43)//n = 42 BD, n = 30 MDD, n = 36 HC	M/I	MDD diagnosis according to DSM-IV or DSM-5 criteria. Severity of depressive or bipolar symptoms assessed by HAM-D or YMRS.	16S rRNA (V3–V4) in serum	Functional pathway analysis
		R	–	*Bacteroidetes* was second highest in MDD, but not in BD and HC. Abundance of *Dialister*, *Prevotella*, *Faecalibacterium, Alistipes, Coryneacteriaceae, Bacteroidales, Tsukamurella* was higher in MDD patients compared to controls, while *Pseudomonas* was lower.	MDD showed lower ascorbate and aldarate metabolism compared to control and BD. BD showed highest vitamin C metabolism.
Romijn et al. (2017)	MDD patients (mean age (years): placebo group 35, probiotic group 36)//8 weeks of intervention//n = 79 (placebo, probiotic treatment)	M/I	Severity of depressive symptoms assessed by MADRS. MDD diagnosis and study inclusion when QIDS-SR ≥ 11 or DASS-42 ≥ 14.	Probiotics with *Lactobacillus helveticus* and *Bifidobacterium longum*.	–
		R	No difference between placebo and probiotic treatment. Both groups reduced depressive symptoms.	No difference between placebo and probiotics.	Vitamin D levels have a moderate effect on treatment; the more vitamin D, the better the improvement under probiotic treatment.
P. Zhang et al. (2023)	Bipolar depression (BD) with a depressive episode (mean age (years): BD normal weight 23, BD obese 26, healthy controls 21)//n = 29 per group of BD patients with and without obesity, n = 31 healthy controls	M/I	MDD diagnosis according to DSM-IV criteria. Severity of depressive symptoms assessed by MADRS and HAMD.	16S rRNA (V3–V4)	–
		R	Higher MDRS and HAMD-24 scores in patients with BD compared to healthy controls.	Compared to control: alpha diversity reduced in patients with obesity, but nut in normal weight patients. *Alcaligenes* was negatively correlated with HAMD. *Finegoldia* negatively and *Collinsella* positively correlated with MADRS.	Obese BD patients’ microbiome showed increased vitamins and cofactors, while for normal wight BD patient's microbiome B6 metabolism was decreased.
Zhao et al. (2022)	MDD patients, drug naive (mean age (years): MDD 30, healthy control 31)//n = 24 MDD patients, n = 26 healthy controls	M/I	MDD diagnosis according to DSM-5 criteria. Severity of depressive symptoms assessed by HAMD, IDS-SR, QIDS-SR.	Whole genome sequencing	Metabolome analysis in blood
		R	–	Differences in the abundance of 94 bacteria between HC and MDD. The ten most abundant species in healthy controls: *Veillonella, Paraprevotella sylaniphila, Bacteroides finegoldii, Mairvinbryatia formatexigens, Massilioclostridium coli, Acetobacter, Facalitalea vylindroides, Prevotella melaninogenicea, Megasphaera* sp *Bacteroides*. The ten most abundant species in MDD patients: *Clostirdium* sp *SS2 1, Deltaproteonacteria bacta g fil, Ruminococcus* sp *CAG 108, Collinsella tanakaei, Ruminococcus bromii, Leuconostoc mesenteroides, Collinsella intestinalis, Bifidobacterium longum, Streptococcus, Eubacterium* sp.	Blood Nicotinamide is one of the highest increased metabolites in MDD patients compared to control and showed altered metabolic pathways. Blood Vitamin A increased in MDD patients. Digestion and absorption were altered for both vitamins. Correlations: Vitamin A and B2 correlated positively with *Ruminococcus* and *Bifidobacteria* species, *Lactococcus, Streptococcus, Lactobacillus, Enterococcus*, as well as *Bacilli* (with some exceptions). While *Clostridia, Streptococcus pyogenes, Faecalitalea, Bacillus flexus, Bacteroides* and *Prevotella* were associated with lowered Vitamin B2. Vitamin A was reduced at higher *Ruminococcus torques* and *Bacteroides pyogenes* abundance.

Note: Articles included after application of inclusion and exclusion criteria on systematic literature search results. Abbreviation: Beck Depression Inventory (BDI), Bipolar Disorder (BD), Center for Epidemiological Studies-Depression scale (CES-D), Colony Forming Units (CFU), Diagnostic and Statistical Manual of Mental Disorders 4^th^ version (DSM-IV), Depression, Anxiety and Stress Scale (DASS-42), Hamilton Depression scale (HAMD), High-Performance Liquid Chromatography (HPLC), Inventory of Depressive Symptoms-Self Report (IDS-SR), Major Depressive Disorder (MDD), Measurement/Intervention (M/I), Patient Health Questionnaire 9 (PHQ-9), Quick Inventory of Depressive Symptomatology-Self Report (QIDS-SR), Result (R), Self-rating Depression Scale (SDS), Montgomery–Åsberg Depression Rating Scale (MADRS).

#### 3.2.1 Animal studies

In rodent models, depressive behavior was induced by alcohol (Jiang et al., 2020), adrenocorticotropic hormone (ACTH) (Song et al., 2019) administration or exposure to chronic unpredictable mild stress (CUMS) (Hao et al., 2021; M. Zhang et al., 2023). These studies showed lowered gut microbiome alpha-diversity and changes in the bacterial taxa abundance in animals with depressive symptoms (Hao et al., 2021; Jiang et al., 2020; Song et al., 2019; M. Zhang et al., 2023). Administration of vitamin B3 reduced depressive symptoms and induced microbiome changes, making it comparable to the control group. Further, FMT from mice treated with/without vitamin B3 into healthy mice, showed a reduction of depressive symptoms after FMT from B3 treated mice (Jiang et al., 2020). Metabolite analysis revealed that vitamin B1, B6 and C metabolism of gut microbiome was enriched after treatment with a plant derived phenolic acid (coniferyl ferulate) with antioxidant properties, which reduced depressive symptoms, compared to CUMS group without treatment (Hao et al., 2021). Altered vitamin digestion and absorption was linked with increased depressive behavior in mice, which could be reversed mostly by antidepressant treatment (M. Zhang et al., 2023). Additionally, vitamin C metabolism pathways were affected by ACTH administration, and a positive correlation for *Akkermansia* and a negative for *Ruminococcus* with vitamin C was reported (Song et al., 2019). These studies indicate that depressive behavior goes along with gut microbiome alterations associated with vitamin metabolism disturbances. Furthermore, they show that negative consequences can be alleviated by improving vitamin supply or microbial vitamin metabolism.

#### 3.2.2 Human studies

In line with the animal studies presented, four of the included human studies pointed towards a beneficial connection between higher vitamin intake and its abundance in blood with MDD symptoms (Li et al., 2022; Maitiniyazi et al., 2022; Mayneris-Perxachs et al., 2022; Rhee et al., 2020) (for details see Table 5). In contrast, increased vitamin abundance and positive correlations between vitamins and depressive symptoms were reported (Li et al., 2022; Mayneris-Perxachs et al., 2022). While in other studies the authors only reported alterations or correlations in the context of MDD (Ciocan et al., 2021; Mayneris-Perxachs et al., 2022; Zhao et al., 2022), which makes a clear interpretation of the study results difficult. Moreover, cofactors that can influence the vitamin metabolism need to be considered, as e.g. Zhang et al. (2023) showed different results in the microbiome’s vitamin metabolism between normal and overweight BP patients.

Furthermore, vitamin consumption altered the microbiome composition. Maitiniyazi et al. (2022) showed that vitamin B2 intake correlated negatively with *Campilobacterota* and *Streptococcus* abundance and positively with *Caldatribacteriota* and *Ruminococcus*. Vitamin B3 intake and *Streptococcus* showed a negative correlation, while it positively correlated with *Ruminococcus* (Maitiniyazi et al., 2022). Additionally, blood vitamin B3 and A concentrations positively correlated with *Bifidobacterium*, which showed increased abundance in MDD patients (Zhao et al., 2022).

Microbial genes involved in vitamin metabolism were less expressed in MDD patients compared to controls (Duan et al., 2022). B2, B5 and B9 synthetase encoding bacteria were higher in healthy controls than in bipolar disorder (BD), while for B6 production microbial species were more abundant in BD patients (Li et al., 2022). *Streptococcu*s was increased in depressed patients (Li et al., 2022; Mayneris-Perxachs et al., 2022; Zhao et al., 2022) and negatively correlated to vitamin B3 production (Maitiniyazi et al., 2022). Li et al. (2022) further demonstrated the connection between microbiome, depressive symptoms and vitamin availability, by showing that approximately 1/8 of the metabolite variance in serum samples and the majority of microbial variance could be explained by the disease status. To sum up, these studies highlight the importance of vitamin-producing gut bacteria in MDD.

Ciocan et al. (2021) showed that antidepressant treatment increased the abundance of *Bacilli,* which was negatively correlated with depression severity. Treatment response was associated with three taxa (*Firmicutes, Tetrasphaera* and *Bosea*) and dependent on blood vitamin metabolism before the treatment started. These results indicate that targeting vitamin and microbiome status of MDD patients, e.g. by probiotics, holds the opportunity to increase treatment response, as already suggested in the literature (Almeida et al., 2015; Liu et al., 2019).

#### 3.2.3 Supplementation of probiotics and vitamins in depression treatment

This section includes 4 human studies addressing probiotic treatment and vitamins in MDD. Two studies showed that a combinatory probiotic and vitamin treatment reduced depressive symptom severity (Feher et al., 2014; Raygan et al., 2018), which was attributable to the probiotics (Feher et al., 2014). In contrast, Reininghaus et al. (2020) reported that MDD symptom reduction was independent from probiotic treatment and intake of vitamins was sufficient for symptom reduction (see Table 4). Whereas vitamin B5 and CoA biosynthesis were shown to be enriched in the microbiome of the probiotic treatment group (Reininghaus et al., 2020). In a randomized controlled trial by Romijn et al. (2017) no difference between probiotic and placebo group were found, but higher blood vitamin D levels were significantly associated with improved depressive symptoms after probiotic treatment (Romijn et al., 2017). These initial studies show mixed results of vitamins or probiotics on MDD symptoms. Nevertheless, we still need to understand the precise mechanisms underlying these intervention effects.

### 3.3 Interconnection between vitamins and mitochondria in depression

The following section presents eight articles with five rodent and three human studies (see Table 6 and Table 7) underlining the importance of vitamins in mitochondrial function.

**Table 6:** Depression, Mitochondria and Vitamin in rodents.

Authors	Study population//Intervention time//n		Depression	Mitochondria	Vitamin
De Oliveira et al. (2009)	Male Wistar rat (290–320g)//28 days//n = 8–10 per group (4 groups: control, 1000, 2500, 4500, 9000 IU/kg vitamin A)	M/I	SPT, FST, TST	Spectrophotometric measurement of superoxide dismutase and catalase activity, lipid peroxidation, oxidative damage of proteins, superoxide production and respiratory chain complex activity were performed in frontal cortex tissue.	1000, 2500, 4500, 9000 IU/Kg/day vitamin A supplementation.
		R	No changes seen for SPT, FST, TST.	Vitamin A supplementation led in the prefrontal cortex to increased lipid peroxidation (for 4500 and 9000 IU/kg/day), and protein damage (for 4500 and 9000 IU/kg/day), superoxide production (for 2000, 4500 and 9000 IU/kg/day), superoxide dismutase activity (for 9000IU/kg/day), complex I and III activity (for 4500 and 9000 IU/kg/day). Complex II and IV and catalase activity were unchanged.	–
T. Liu et al. (2020)	Male C57Bl/6 mice (weight 18–22g)//21 days, Chrysanthemum morifolium (Chr), Naringenin (Nar) and apigenin (Api) or fluoxetine//n = 48 (6 groups: control, corticosterone (20 mg/kg), corticosterone + fluoxetine (5 mg/kg), corticosterone + Chr (3.33 g/kg), corticosterone + Nar (100 mg/kg), corticosterone + Api (20 mg/kg))	M/I	Depression induction via corticosterone injection. SPT	Metabolome pathway analysis of urine samples.	Metabolome pathway analysis or urine samples.
		R	Decreased SPT after corticosterone treatment. Chrysanthemum morifolium, Naringenin and apigenin and fluoxetine increased SPT.	Changes in citrate cycle.	Changes in nicotinate and nicotinamide metabolism. Nicotinuric acid was lowered by corticosterone but could be increased by Chrysanthemum morifolium, apigenin and fluoxetine.
Moretti et al. (2013)	Female Swiss mice (35–45g)//vitamin or water injection 1 h before restrained stress//n = 32 (4 groups: Control, unstressed with vitamin C, Stressed with or without vitamin C)	M/I	7 h restrained stress, FST.	Cortex and hippocampal tissue. Photometrical determination of glutathione levels, glutathione reductase, glutathione peroxidase, superoxide dismutase activity and lipid peroxidation.	Ascorbic acid (1 mg/kg)
		R	Mice showed more immobility after stress, but ascorbic acid and Fluoxetine administration led to decreased immobility after the stressor.	In cerebral cortex and hippocampus increased lipid peroxidation and superoxide dismutase activity was seen after stressor. Vitamin C reversed these increases to normal levels. Glutathione levels were not affected by the stressor or vitamin. Vitamin C never showed changes in non-stressed mice. In cerebral cortex increased glutathione reductase and glutathione peroxidase was found after stressor, which could be reduced to normal level by vitamin C administration.	–
Motafeghi et al. (2022)	Wistar rats (age: 1 day after birth)//Stressors; Starting at day 60 rats received daily treatment with medication for 14 days.//n = 80 (8 groups: control group, maternal separation, maternal separation and treatment with 5-methyltetrahydrofolate (5MTHF) or citalopram (20 mg) or edaravone and minocycline (1, 20, 50 mg) or citalopram + edaravone and minocycline)	M/I	Maternal separation between postnatal day 1 and 14. Then till day 21 next to their mothers. From day 21 the rats were separated from mothers. At day 28, 35 and 60 rats received 2h of restrained stress and 20min forced swim stress. Measured with TST, SPT and FST at day 74.	ROS production, mitochondrial membrane potential.	3 mg/kg 5-methyl-tetrahydrofolate (5MTHF) (Vitamin B9)
		R	In the TST and FST the stressed group showed longer immobility compared to control. The increased immobility could be significantly lowered by 5MTHF supplementation. Sucrose intake was lowered in the stressed group but could be reversed by in 5MTHF.	ROS production and mitochondrial membrane potential were lowered in the stressed group compared to 5MTHF. Mitochondrial function and GSH were higher in stressed rats compared to stressed rats with 5MTHF.	–
Tagliari et al. (2010)	Male Wistar rats (age: 60 days)//40 days of chronic variable stress with or without daily vitamin administration//n = 5–6 animals per group (4 groups: control, stressed, Vitamin, Stress + vitamin)	M/I	FST	Spectrophotometric measurement of cytochrome *c* oxidase and Complex II activity in hippocampus and/or in prefrontal cortex tissue.	Vitamin E (40 mg/kg), vitamin C (100 mg/kg)
		R	Stressed rats showed increased immobility.	Chronic variable stress showed a reduction on cytochrome *c* and complex IV activity in prefrontal cortex a hippocampus and a reduction of complex II activity in prefrontal cortex. All effects were reversed by Vitamin C and E administration. No differences were shown in the pyruvate kinase activity.	–

*Note:* Articles included after application of inclusion and exclusion criteria on systematic literature search results. Abbreviation: Forced Swim Test (FST), Measurement/Intervention (M/I), 5-Methyltetrahydrofolate (5MTHF), Result (R), Sucrose Preference Test (SPT), Tail Suspension Test (TST).

**Table 7:** Depression, Mitochondria and Vitamin in humans.

Authors	Study population//Intervention time//n		Depression	Mitochondria	Vitamin
De la Fuente et al. (1998)	Aged women (mean age (years): 72)//16 weeks, daily vitamin supplementation//n = 30 (three groups: healthy controls, MDD, coronary heart disease)	M/I	MDD diagnosis according to DSM-III criteria and severity of depressive symptoms assessed by SCID-UP interview.	Spectrophotometric measurement of superoxide production in neutrophils and lipid peroxide levels in serum.	Vitamin C (1 g), vitamin E (200 mg)
		R	MDD group had highest lipid peroxidation and superoxide production.	Vitamin supplementation led to lowered lipid peroxide levels and superoxide production in all groups. MDD was as high as control after treatment.	–
Mocking et al. (2021)	Remitted recurrent MDD and healthy controls (mean age (years): Women with MDD 27, Men with MDD 25, HC age and sex matched)//n = 127 (in four groups: n = 45 women and n = 23 men with MDD; n = 40 healthy women, n = 19 healthy men)	M/I	MDD diagnosis according to DSM-IV criteria and severity of depressive symptoms assessed by SCID and HAMD.	Metabolome analysis in plasma.	Metabolome analysis in plasma.
		R	–	In MDD patients lowered acyl-carnitines and cardiolipins were found, which are markers for mitochondrial membrane complexity, biomass and fatty acid oxidation.	Decreased B2 metabolism in MDD compared to control. In men higher β-carotene levels were seen in men and were associated with a longer time until recurrence. Men showed decreased vitamin B1, B6 and vitamin E metabolites compared to healthy controls.
Zheng et al. (2016)	MDD patients and healthy controls (mean age (years): MDD 39, HC 40)//n = 100 (n = 50 MDD, n = 50 HC)	M/I	MDD diagnosis according to DSM-IV criteria. Severity of depressive symptoms assessed by HAMD.	Metabolome analysis in PBMC.	Metabolome analysis in PBMC.
		R	–	Malate, fumarate, sorbitol and ribulose-5- phosphate were lower in MDD compared to HC.	Less vitamin E in PBMCs of MDD patients compared to HC

*Note:* Articles included after application of inclusion and exclusion criteria on systematic literature search results. Abbreviation: Diagnostic and Statistical Manual of Mental Disorders 3^rd^/4^th^ version (DSM-IIII/IV), Hamilton Depression scale (HAMD), Major Depressive Disorder (MDD), Measurement/Intervention (M/I), Result (R), Structured Clinical Interview for Diagnostic (SCID).

In mice, induction of depressive-like behavior with corticosterone injection revealed alterations in citrate cycle metabolites and vitamin B3 metabolism in urine samples (T. Liu et al., 2020). 5-methyltetrahydrofolate (5MTHF) – the active form of vitamin B9 – showed a reduction of depressive behavior in stressed rats comparable to rats treated with citalopram. Furthermore, 5MTHF as well as citalopram reduced ROS production, increased glutathione levels and lowered mitochondrial membrane potential in rat brains (Motafeghi et al., 2022). De Oliveira et al. (2009) investigated the effects of vitamin A supplementation in rats, resulting in increased prefrontal cortex lipid peroxidation, protein damage, superoxide production, superoxide dismutase activity, and complex I – III activity, while no increase in depressive-like behavior was observed (De Oliveira et al., 2009). Combinatory vitamin C and E treatment during a chronic variable stress period in rats, showing increased depressive-like symptoms, prevented cytochrome *c* and complex II activity reduction in the brain, while pyruvate kinase activity was not affected (Tagliari et al., 2010). Vitamin C administration reversed stress-induced increase in lipid peroxidation, cellular oxidative stress defense mechanisms in mouse brains and reduced depressive-like behavior in the FST to a level comparable to unstressed mice (Moretti et al., 2013).

Similar effects could be seen in humans, as De la Fuente et al. (1998) reported severely increased superoxide production and lipid peroxide levels in women with MDD, which were lowered to the same level as observed in the healthy control group after sixteen weeks of vitamin C and E intake. Vitamin E as well as several energy metabolites in PBMCs were reduced in patients with MDD compared to healthy controls (Zheng et al., 2016). Mocking et al. (2021) reported lowered FAD^+^, vitamin B2 levels and biomarkers for mitochondrial biomass and complexity in both sexes. A higher risk of MDD recurrence with lower plasma vitamin A levels and lower vitamin B1, B6 and E metabolites were observed in men but not in women (Mocking et al., 2021).

These results confirmed the high impact of vitamin B and antioxidant vitamins like C and E on mitochondrial functions in MDD.

### 3.4 Interconnection between gut microbiome, mitochondria, and vitamins

This final section, which includes four articles, summarizes microbiome and mitochondria interaction, and thus underlines the potential of vitamins produced by the microbiome as mediators of this connection (see Table 8 and Table 9).

**Table 8:** Mitochondria, Microbiome and Vitamins in rodents.

Authors	Study population//Intervention time//n		Microbiome	Mitochondria	Vitamin
Ge et al. (2022)	C57BL/6 mice, pubs of females with or without B12 supply during pregnancy//pregnant mice had 21 days of B12 abbreviation or supplementation. Further 1,5, or 8 weeks with or without B12 after birth till scarification.//n = 3–5 per group, depending on the analysis	M/I	16S rRNA (V4–V5)	Gene expression, metabolomics, seahorse oxygraph	B12 (400 ng/twice weekly after weaning, 40 ng/twice weekly before weaning)
		R	B12 deficiency impaired complexity of microbiome mice in the age of 8 weeks. In ileum, cecum and colon of B12 supplemented animals showed higher *Turicibacter, Lactobacillus, Bacterioides* and decreased *Allobaculum* compared to B12 deficient mice. In the colon reduced *Lachnospiraceae* and in cecum reduced *Coprococcus* was found in vitamin B12 deficient mice.	Increased abundance OXPHOS related genes in B12 supplemented mice. Less citrate acid cycle, β-oxidation intermediates and other B vitamins. Seahorse analysis showed that vitamin B12 deficiency leads to lower basal respiration.	–
Kim et al. (2023)	Male C57BL/6 mice (age: 6 weeks)//35 days//n = 49 (10 controls and 13 for each experimental group); CT26- induced cancer cachexia (CC), CC+ β-carotene (0.5 mg/kg body wight), CC+ β-carotene (2 mg/kg body wight)	M/I	16S rRNA (V4)	Extracellular acidification rate and oxygen consumption rate via seahorse in adipocytes. Lactate, glucose and ATP concentrations in cultured subcutaneous adipocytes. Liver and subcutaneous fat mRNA expression.	β-carotene supplementation
		R	Alpha diversity was lowered by β-carotene supplementation. β-diversity was reduced in the CC group and restored to control comparable values by β-carotene. CC compared to control showed lower *Lactobacillus, Eubacterium coprostanoligenes* group*, Intestinimonas, Enterorhabdus, Clostridia vadinBB60* group and *Turicibacter* but higher *Blautia*. β-carotene restored *Eubacterium* C*oprostanoligenes* group, *Intestinimonas*, *Clostridia vadinBB60* group and *Turicibacter*, making it comparable to control.	Upregulated mRNA of brown fatty tissue (Ucp1, Pdk4) in mice with tumor, was reversed by β-carotene administration. Lower basal respiration and proton leak and higher spare respiratory capacity in CT26 treated preadipocytes compared to control. Proton leak and basal respiration were restored by 1 μM β-carotene supplementation. β-carotene concentrations above 20 μM reduced oxygen consumption rate in colon cancer cells.	–
Wu et al. (2022)	Male Sprague-Dawley rat (age: 4 weeks)//probiotic treatment with or without diquat treatment//n = 60 (10 per group: saline ± Diquat (DQ) injection, Probiotic *Bacillus amyloliquefaciens* ± Diquat (DQ), *Bacillus licheniformis* ± Diquat (DQ))	M/I	16S rRNA sequencing, metagenomic sequencing. 24 days of *Bacillus amyloliquefaciens SC06* or *licheniformis SC08* supplementation. Oral administration of 10ˆ7 CFU/ml. At day 24 diquat (oxidative stress inducing agent). End of experiment after 26 days.	Membrane potential and mitochondrial structure.	Pathway analysis
		R	Superoxide dismutase positively correlated with *Butyricicoccus, Faecalibacterium, Akkermansia* and negatively correlated with *Bifidobacterium, Shigella abundance*. Positive correlation was seen between MDA and *Butyricimonas*, negative with *Lactococcus, Akkermansia, Escherichia-shigella*.	Probiotic treatment decreased oxidative damage, cytochrome *c* release and ROS production after diquat treatment, but had no effect on untreated rats. Mitochondrial membrane potential reduction after diquat treatment was restored by SC06. Antioxidant capacity and superoxide dismutase was decreased by diquat. SC06 increased Superoxide dismutase activity.	SC06 and SC08 treatment reduced vitamin B7 metabolism and ubiquinone pathways. SC06 reduced B1, B2, B3, B6 and B9 metabolism of microbiome.

*Note*: Articles included after application of inclusion and exclusion criteria on systematic literature search results. Abbreviation: Adenosine Triphosphate (ATP), Cancer Cachexia (CC), Colony Forming Units (CFU), Diquat (DQ), Measurement/Intervention (M/I), Oxidative Phosphorylation (OXPHOS), Reactive Oxygen Species (ROS), Result (R).

**Table 9:** Mitochondria, Microbiome and Vitamins in humans.

Authors	Study population//n		Microbiome	Mitochondria	Vitamin
Chang et al. (2019)	PBMC isolation from healthy donors, publicly available Dataset of microbiome in Crohn's disease//n = 2 healthy donors	M/I	Publicly available dataset from the National Center for Biotechnology Information.	Seahorse in PBMC (2 weeks culture in Th17 polarizing conditions with different metabolites identified from the Crohn's disease database).	Vitamin C (100 μM)
		R	–	*Pseudomonas* is capable of vitamin C production via 2,5-diketo-gluconic acid pathway. *Erwinia, Serratia, Roseomonas Yersinia* and some *Azorhizophilus bacteria* have genes to perform this pathway, too.	Vitamin C reduced activated Th17 cells and inhibited cytokine production to more than half. No reduction of IL-10 producing cells.

*Note*: Articles included after application of inclusion and exclusion criteria on systematic literature search results. Abbreviation: Measurement/Intervention (M/I), Peripheral Blood Mononuclear Cells (PBMC), Result (R).

Using a B12 deficient mouse model, Ge et al. (2022) showed that mitochondrial basal respiration, microbiome complexity, expression of genes involved in the OXPHOS and metabolites of the citrate cycle, β-oxidation and other B vitamins were reduced and bacterial abundance was altered compared to mice provided with B12 (for detailed information see Table 8). In a mouse model of cancer cachexia, β-carotene – a vitamin A precursor – administration reversed changes in preadipocyte mitochondrial basal respiration, proton leak, spare respiratory capacity, microbial alpha diversity, beta diversity and abundance of specific bacteria to a level comparable to controls (Kim et al., 2023). *Bacillus* supplementation protected rats from oxidative stress induced by the herbicide diquat and restored mitochondrial membrane potential. Tested strains reduced vitamin B metabolism of the gut microbiome. A positive correlation of superoxide dismutase and *Akkermansia* were found and oxidative stress was reduced the more *Lactococcus* and *Akkermansia* were present (Wu et al., 2022). Several bacteria have the potential for de-novo synthesis of vitamin C and in humans vitamin C has shown to dampen mitochondrial respiration in activated T cells, while not affecting resting T cells (Chang et al., 2019) (for details see Table 9).

Taken together, these study results indicate that there is a bidirectional connection between gut bacteria and vitamins, affecting each other and mitochondrial activity.

## 4 Discussion

In sum there is substantial evidence for an interaction between the microbiome and mitochondria as well as the positive effects of a probiotic treatment on depressive symptom severity, which might be mediated by vitamin production. Unfortunately, to the best of our knowledge, there is no single article addressing all four topics (mitochondria, microbiome, vitamins and depression) within a single study. This might hold the risk of misinterpretation of the vitamin interaction between mitochondria and microbiome. Nevertheless, by examining the underlying connections of the individual topics this systematic review found strong indications of a role of vitamins mediating the connection of the microbiome and mitochondria in the context of depression. Therefore, evidence-based hypotheses should be tested to address this research gap, to improve our knowledge and to develop supportive therapy concepts to increase the success of currently available treatment options. It is already shown that vitamins could improve depressive symptoms and therapy success.

### 4.1 Effects of B vitamins in depression and therapy success

Therapy success in patients with depression was predicted by vitamin B9 level (Fava et al., 1997) and associated with *Firmicutes* (Ciocan et al., 2021) – capable of vitamin B9 production (Rudzki et al., 2021). Lowered vitamin B9 and B12 levels were found in therapy resistant patients (Coppen and Bolander-Gouaille, 2005). Insufficient vitamin B2 and B3 metabolism prevents pharmacotherapy treatment success (Ciocan et al., 2021). Most B complex vitamins were found to be lower in patients with depression compared to healthy controls (Li et al., 2022; Maitiniyazi et al., 2022; Mocking et al., 2021). Additionally, there are negative associations between the production of vitamins by bacteria or vitamin intake and depressive symptoms (Li et al., 2022; Mayneris-Perxachs et al., 2022; Reininghaus et al., 2020). Data on therapy advantages of B9 and B12 supplementation compared to placebo or in combination with antidepressant medication is very inconsistent, although a long-term use could be more efficient compared to short term application (see review: Almeida et al., 2015; Markun et al., 2021). Contrasting positive effects of vitamins on MDD, some studies reported a positive correlation of vitamin B1 and B7 intake and microbial metabolism with depressive symptoms (Mayneris-Perxachs et al., 2022), as well as increased vitamin B2 (Zhao et al., 2022) or B6 (Li et al., 2022) levels in patients with depression. Meta-analytic results show that vitamin supplementation alone has no antidepressive effect or shows only small effects (Vellekkatt and Menon, 2019; Young et al., 2019).

### 4.2 Effects of vitamin A in depression and therapy success

Vitamin A is another vitamin to be considered critically. High levels of vitamin A have negative effects on mood (Zhao et al., 2022), ROS production and oxidative stress (De Oliveira et al., 2009), while low levels are beneficial for treatment response (Ciocan et al., 2021). This fits to literature, as vitamin A activates the HPA axis and was already shown to induce depressive symptoms, while vitamin A antagonists prevent symptom development in mice. On the one hand, vitamin A influences multiple neurotransmitters by changing the gene expression of neurotransmitter receptors, degrading enzymes or reuptake transporters (see review: Hu et al., 2020). On the other hand, low levels of β-carotene (a precursor of vitamin A) decrease the risk of recurrence, nevertheless this was only found in men (Mocking et al., 2021). Vitamin A reduced depressive symptoms during chronic inflammation in humans (Bitarafan et al., 2016) and rescued altered mitochondrial functions in mice (Kim et al., 2023). Differences could be explained by microbial abundance. For example, *Bifidobacterium bifidum* has been shown to be able to turn vitamin A into its active form (Woo et al., 2021). Maitiniyazi et al. (2022) reported low *Bifidobacterium* abundance in MDD patients and showed that a high vitamin A intake went along with a lower depressive scores. In contrast Zhao et al. (2022) reported high *Bifidobacterium* abundance correlating with high vitamin A levels, both increased in MDD patients.

### 4.3 Effects of vitamins C, D and E in depression and therapy success

The studies included here on vitamin C and E underline their positive effect on superoxide production and lipid peroxidation (De la Fuente et al., 1998; Moretti et al., 2013; Tagliari et al., 2010). Both vitamins can be produced by the microbiome (Chang et al., 2019). Negative correlations of vitamin C (Rhee et al., 2020; Song et al., 2019) and vitamin E (Mayneris-Perxachs et al., 2022) with depression severity were reported. Such a result is not surprising when taking the link between increased vitamin levels and improved mitochondrial respiration into consideration (for details see 4.4). For vitamin D and E supplementation the majority of the results point towards a positive effect of treatment (Casseb et al., 2019; Lee et al., 2022) and changes in the abundance of bacteria (Choi et al., 2020; Pham et al., 2021; Waterhouse et al., 2019).

Taken together, both vitamins and gut bacteria can influence (mental) health and therapy success. Therefore, closer attention needs to be paid to gut bacteria and their vitamin metabolism, especially in the context of mitochondrial energy production in MDD.

### 4.4 Modulation of mitochondrial function in depression by the microbiome and vitamins

Impaired mitochondrial energy production in depression was already shown in blood immune cells, platelets and muscle cells (Gardner, 2003; Karabatsiakis et al., 2014; Zvěřová et al., 2019). Latest research is pointing to a modulatory impact of the gut microbiome and its metabolites on mitochondrial function. The literature presented here showed that FMT from patients with depression into healthy rodents affected brain mitochondrial protein expression and changes gut mitochondrial morphology (Liu et al., 2021; S. Liu et al., 2020). The gut microbiome can have both negative and positive effects on mitochondrial function and depression, Lukić et al. (2019) showed that *Ruminococcus flavefaciens* was linked to changes in protein-protein interactions of mitochondrial respiratory complexes, while abolishing effects of pharmacologic antidepressant treatment. In contrast, probiotic treatment lowered depressive symptoms and went along with lowered ROS abundance in patients showing higher ROS abundance before treatment (Venturini et al., 2019). A positive correlation of superoxide dismutase with *Akkermansia* was shown (Wu et al., 2022), while lowered *Akkermansia* abundance was already shown in depression (Aatsinki et al., 2020; McGaughey et al., 2019). *Faecalibacterium* positively correlated with superoxide dismutase (Wu et al., 2022) and is thought to be reduced in MDD (Li et al., 2022; McGuinness et al., 2022).

In several rodent studies it could be shown that changes in the gut microbiome or vitamin abundance went along with mitochondrial changes in the brain (Liu et al., 2021; Lukić et al., 2019; Moretti et al., 2013; Motafeghi et al., 2022; De Oliveira et al., 2009; Tagliari et al., 2010; Wang et al., 2022). The manifestation of depressive behavior might be the result of a systemic energy deficiency e.g. due to reduced vitamin abundance, which is having the highest impact on neurons, as other cells can produce energy by mitochondria-independent pathways (Bolaños et al., 2010). Corroborating the importance of vitamin E and its microbial production on mitochondrial function (Wesselink et al., 2019), it was shown that Vitamin C and E administration prevented stress induced reduction of cytochrome *c* and complex II activity in the rat brain (Tagliari et al., 2010). In MDD patients vitamin E and energy metabolism was reduced in PBMCs (Zheng et al., 2016). Furthermore, vitamin C dampens mitochondrial respiration in activated T-cells, while not affecting resting T cells (Chang et al., 2019). Beneficial effects of vitamin C supplementation on depressive symptoms (Naylor and Smith, 1981) further underline the importance of vitamins on mitochondrial function in the context of MDD. Lowered FAD^+^, vitamin B2 levels, and biomarkers for mitochondrial biomass and complexity were observed in patients with MDD (Mocking et al., 2021). B12 deficiency results in lowered OXPHOS gene expression and basal respiration in mice (Ge et al., 2022). Vitamin A reduced depressive symptoms during chronic inflammation in humans (Bitarafan et al., 2016) and protected against changes in mitochondrial function in mice (Kim et al., 2023). Nevertheless, vitamin A increases ROS production, protein damage, lipid peroxidation, and Complex I - III activity (De Oliveira et al., 2009), which in turn can lead to even more ROS production (Mazat et al., 2020). Contradictory results should encourage future research to elucidate the complex interconnection between mitochondria and vitamins.

The literature presented indicates positive effects of the gut microbiome and (microbiome-derived) vitamins. Therefore probiotic supplementation as a new treatment approach of depression holds a high potential to improve therapy success.

### 4.5 Probiotic supplementation for depression treatment

Existing literature shows that probiotic treatment often includes bacterial species capable of vitamin production (see review: R. T. Liu et al., 2019). The studies included in this review (n = 5) used *Lactobacillus casei* and *Bifidobacterium bifidum* (Akkasheh et al., 2016; Raygan et al., 2018; Venturini et al., 2019); *Bifidobacterum longum* (Romijn et al., 2017; Venturini et al., 2019); *B. infantis* (Venturini et al., 2019); *Lactobacillus plantarum* (Reininghaus et al., 2020)*, Lactobacillus acidophilus, L. reuteri* and *L. fermentum* (Raygan et al., 2018). Bacterial strains used in these studies are linked to producing vitamin B1, B2, B6, B9 and B12 (Yoshii et al., 2019). In contrary, a study by Romijn et al. (2017) couldn’t report positive effects of probiotics compared to placebo, even though *Bifidobacterium longum* (capable of B6 and B12 production) was used (Romijn et al., 2017; Yoshii et al., 2019). A possible explanation could be that probiotic use of *Bifidobacterium longum* might even be disease promoting, as its abundance was reported to be higher in MDD patients (Zhao et al., 2022), thus warranting further investigation on its effect on MDD symptoms. Therefore, a better understanding of the different bacteria and their role is necessary to design a safe and effective treatment by probiotic supplementation in MDD.

### 4.6 The role of gut microbial vitamin production in depression

Besides the application of probiotics, the beneficial gut bacteria are linked to health promoting effects. *Akkermansia* correlates positively with vitamin C and B2, B5, B9 synthesis (Li et al., 2022; Song et al., 2019) and showed protective effects from oxidative stress (Wu et al., 2022). *Akkermansia* abundance was reported to be negatively correlated with depressive symptoms (Aatsinki et al., 2020; McGaughey et al., 2019) even though increased *Akkermansia* abundance was seen in mice with depression, which might be due to the method of alcohol supplementation to induce depressive symptoms (Jiang et al., 2020). *Streptococcus* was shown to be increased in depressed individuals (Li et al., 2022; Mayneris-Perxachs et al., 2022; Zhao et al., 2022) and is negatively correlated with vitamin B3 production (Maitiniyazi et al., 2022), in accordance with systematic review results (McGuinness et al., 2022; Simpson et al., 2021). For *Bacteroides,* study results are very heterogenous (McGuinness et al., 2022), corresponding to results in this review; reporting two studies (Li et al., 2022; Maitiniyazi et al., 2022) with increased *Bacteroides* in MDD and two studies with decreased abundance (Hao et al., 2021; Zhao et al., 2022). *Escherichia-Shigella* are known as opportunistic pathogens and their abundance was higher in depressed individuals in two studies included (Maitiniyazi et al., 2022; Rhee et al., 2020) and were positively correlated with oxidative stress markers (Wu et al., 2022). *Ruminococcus* was negatively correlated with Vitamin C (Rhee et al., 2020; Song et al., 2019) but positively associated with vitamin B2 and B3 intake (Maitiniyazi et al., 2022) and can produce several B-vitamins (Yoshii et al., 2019). Overall *Ruminococcus* is expected to be lowered in MDD patients (Li et al., 2022; McGuinness et al., 2022), while in an animal study it was shown to be higher after ACTH injection (Song et al., 2019) and *R. bromii* was higher in MDD patients (Zhao et al., 2022). These findings underline the importance of gaining a deeper knowledge on the different bacteria species, as bacteria of the same family and even in the same genus might have different functions. Therefore, a closer look on the genera and species might be of high importance for upcoming research.

### 4.7 Potential factors influencing vitamin homeostasis in depression

Considering the sparse knowledge on the diverse vitamin metabolism and its role in mental health, we put forth potential influencing factors involved with vitamin homeostasis in patients with depression:

• A lowered dietary vitamin intake (Nguyen et al., 2017; Prohan et al., 2014; Tolmunen et al., 2003).
• A reduced vitamin production or absorption in the gut e.g. as reported for vitamin B2, B5, B9 (Li et al., 2022).
• An increased vitamin consumption by gut bacteria for their own needs. Therefore, an increase in vitamin metabolism in the microbiome can affect the vitamin availability to the human cells negatively. This hypothesis might help to explain findings of reduced vitamin B1, B2, B3, B6, B7 and B9 metabolism in gut bacteria showing positive effects on mitochondria (Wu et al., 2022).
• Vitamin production and microbiome abundance can be altered by several parameters, e.g., differences in microbial vitamin metabolism were seen between normal weight and obese bipolar depressive disorder patients (P. Zhang et al., 2023).
• In addition to bacteria, the gut is also colonized by fungi, protozoa, archaea and viruses, creating a complex ecosystem termed “biofilm” (Barko et al., 2018). Therefore, it could be of importance to look on the connections between different members of these biofilms to gain knowledge about mutual interference possibly affecting vitamin production.
• Disturbances of the vitamin metabolism can result in an accumulation of certain metabolites. Accumulation might have been shown by Li et al. (2022) as higher levels of vitamin B6 were reported to be positively associated with depressive symptoms, while its catabolic products were reduced (Li et al., 2022).

Therefore, the articles reviewed here on the microbiome, vitamins and their interactions press on the need to address the gut bacteria and vitamin metabolites in patients with depression more precisely. Related research investigating the interaction of mitochondria and microbiome via vitamins in depression would be of high innovation value. Such an integrative approach could yield knowledge to predict treatment response and increase therapy success by applying vitamins and/or probiotic supplementation in the treatment for mental health.

## 5 Conclusion

The studies presented emphasize a beneficial effect of microbiota-based interventions on mitochondria and depression, with vitamins as a possible modulator of this effect. As our literature search did not find all four topics – vitamins, mitochondria, microbiome, and depression – addressed within a single study, this systematic literature search bundled the knowledge currently available and underlines the research gap to encourage new investigations. Taken together, evidence is pointing towards an improvement in depressive symptoms by increasing blood vitamin concentrations or by higher gut microbial vitamin production, and this is linked to an improved mitochondrial energy production. Additionally, antioxidative properties of vitamin C and E prevent extensive ROS production and thereby reduced the negative effects of ROS on mitochondrial function and depression. A closer investigation of the involvement of the microbiome’s vitamin metabolism in vitamin availability as well as on the mediation of the microbiome-mitochondria interaction by vitamins are needed to draw clear conclusions regarding vitamins’ involvement in depression. Therefore, the final conclusion of this review is to emphasize the urgent need to investigate the interplay between microbiome, mitochondria and vitamins in depression. Such knowledge holds the potential to revolutionize current treatment approaches, for instance through the inclusion of vitamin supplementation in treatment strategies to improve therapy success in patients with depression.

## CRediT authorship contribution statement

**Ellen Bisle:** Conceptualization, Data curation, Methodology, Visualization, Writing – original draft, Writing – review & editing. **Suchithra Varadarajan:** Conceptualization, Writing – review & editing. **Iris-Tatjana Kolassa:** Conceptualization, Funding acquisition, Supervision, Writing – review & editing.

## Declaration of competing interest

The authors declare that they have no known competing financial interests or personal relationships that could have appeared to influence the work reported in this paper.

## Data Availability

No data was used for the research described in the article.
