# Supplementary material for: Vitamin-mediated interaction between the gut microbiome and mitochondria in depression: A systematic review-based integrated perspective
Source: Brain Behav Immun Health. 2024 May 10;38:100790. doi: 10.1016/j.bbih.2024.100790 (PMC11225645; doi:10.1016/j.bbih.2024.100790)
Supplement: Multimedia component 1 [file mmc1.docx]

Supplementary material

*Supplementary Table 1 Systematic review centre for laboratory animal experimentation (SYRCLE) assessing bias.*

|  | D, Ba, Mi | | | | D, Mi, V | | | | D, Ba, V | | | V, Ba, Mi | | | | |
| --- | --- | --- | --- | --- | --- | --- | --- | --- | --- | --- | --- | --- | --- | --- | --- | --- |
|  | (Liu et al., 2021) | (S. Liu et al., 2020) | (Lukić et al., 2019) | (Wu et al., 2022) | (Hao et al., 2021) | (Jiang et al., 2020) | (Song et al., 2019) | (M. Zhang et al., 2023) | (Wu et al., 2022) | (Ge et al., 2022) | (Kim et al., 2023) | (Oliveira et al., 2009) | (T. Liu et al., 2020) | (Moretti et al., 2013) | (Tagliari et al., 2010) | (Motafeghi et al., 2022) |
| 1 Was the allocation sequence adequately generated and applied? | U | Y | Y | Y | Y | Y | Y | Y | U | U | Y | U | Y | U | Y | U |
| 2 Were the groups similar at baseline or were they adjusted for confounders in the analysis? | Y | Y | U | U | Y | Y | Y | Y | Y | U | Y | Y | Y | Y | Y | Y |
| 3 Was the allocation adequately concealed? | U | U | U | U | U | U | U | U | U | U | U | U | U | U | U | U |
| 4 Were the animals randomly housed during the experiment? | N | U | U | U | U | U | U | U | U | U | U | U | U | U | U | U |
| 5 Were the caregivers and/or investigators blinded from knowledge which intervention each animal received during the experiment? | N | N | U | U | U | U | Y | U | U | U | U | U | U | U | U | U |
| 6 Were animals selected at random for outcome assessment? | N | Y | U | U | U | U | U | U | U | U | U | U | U | U | U | U |
| 7 Was the outcome assessor blinded? | U | U | U | U | U | U | U | U | U | U | U | Y | U | Y | U | U |
| 8 Were incomplete outcome data adequately addressed? | N | Y | N | Y | Y | Y | N | N | N | N | Y | N | Y | N | N | Y |
| 9 Are reports of the study free of selective outcome reporting? | Y | Y | N | Y | Y | Y | Y | Y | Y | Y | Y | Y | Y | Y | Y | Y |
| 10 Was the study apparently free of other problems that could result in high risk of bias? | N | N | N | U | U | Y | U | U | U | U | N | U | Y | N | N | N |

*Note*: depression (D), microbiome (Ba), mitochondria (Mi) vitamins(V), yes (Y) indicates a low risk of bias, no (N) indicates a high risk of bias, unclear(U) indicates that the risk of bias could not clearly been accessed. This table is based on the instructions by Hooijmans et al. 2014.

Reference:

Hooijmans, C.R., Rovers, M.M., Vries, R.B. de, Leenaars, M., Ritskes-Hoitinga, M., Langendam, M.W., 2014. SYRCLE’s risk of bias tool for animal studies. BMC Medical Research Methodology 14, 43. https://doi.org/10.1186/1471-2288-14-43
